# Global research and emerging trends in depression in lung cancer: a bibliometric and visualized study from 2014 to 2024

**DOI:** 10.3389/fonc.2025.1490108

**Published:** 2025-03-05

**Authors:** Weilan Lin, Shun Chen, Jiawei Chen, Chune Wang, Feng Lu

**Affiliations:** ^1^ Respiratory Department, The Second Affiliated Hospital of Fujian University of Traditional Chinese Medicine, Fuzhou, China; ^2^ First Clinical College, Fujian University of Traditional Chinese Medicine, Fuzhou, China; ^3^ Clinical Research Center for Integrative Medicine on Early Lung Cancer Diagnosis and Treatment of Fujian Province, Fuzhou, China

**Keywords:** lung cancer, depression, visualization, bibliometric, CiteSpace, hotspots

## Abstract

**Background:**

The impact of depression on the physical and psychological well-being of lung cancer patients has raised significant concerns. This study explored lung cancer-related depression research from a bibliometric perspective.

**Methods:**

Articles and reviews on depression in lung cancer published from 2014 to 2024 in the Web of Science Core Collection were retrieved and identified. The information extracted included “Full Record and Cited References”. Bibliometric analysis was conducted using CiteSpace and Excel to examine relevant publications in terms of country, institution, authorship, journal, citations, and keywords.

**Results:**

A total of 761 publications on depression in lung cancer were identified for analysis, contributed by 4,667 authors from 305 institutions and 58 countries. The annual publication count has steadily increased, culminating in a peak in 2024. China (275 papers), Harvard University (58 publications), and Joseph A. Greer (25 publications) were the most productive and influential country, institution, and author, respectively. Jennifer S. Temel emerges as a highly influential author, securing the second position in terms of both publication count and citation impact. The journal with the highest number of publications is Supportive care in cancer, while the Journal of Clinical Oncology has garnered the most citations. Reference and keyword analysis indicated that the research hotspots mainly included 1) Epidemiology of lung cancer-related depression; 2) The deleterious impact of depression on lung cancer patients, particularly with regards to their quality of life; 3) Association between inflammation and depression in lung cancer; 4) Treatment.

**Conclusions:**

Our study employed bibliometric analysis to identify prevalent focal areas and emerging trends in the field of research on lung cancer-related depression. Our study found that numerous unanswered questions persist, including the intricate relationship between lung cancer and depression, the profound impact of depression on lung cancer patients, and the interplay between inflammation and depression in this specific population. Furthermore, a current challenge in clinical practice involves the development of secure and more efficacious treatment strategies for individuals with lung cancer-related depression. These findings provide valuable guidance for scholars seeking to explore new avenues of investigation.

## 1 Introduction

Lung cancer, being the most prevalent neoplasm globally and the leading cause of cancer-related mortality (1), has demonstrated a consistent upward trend in morbidity in recent years (2). The absence of specific clinical symptoms in the early stages of lung cancer poses challenges for early diagnosis, resulting in a majority of patients being diagnosed at advanced stages and missing the optimal window for surgical intervention (3, 4), thereby leading to a low survival rate. Despite advancements in medical research, the overall 5-year survival rate for lung cancer remains below 20% (3). Lung cancer patients not only experience physical symptoms and suffer from adverse effects caused by surgical interventions and chemotherapy, but also face psychological disturbances such as anxiety, fear, and depression (5, 6).

The prevalence of depression is higher in lung cancer patients compared to those with other types of malignancies (7). Both lung cancer itself and tumor stage independently contribute to the development of depression in patients with lung cancer (7, 8); conversely, depression not only acts as an independent risk factor for the onset of lung cancer (9) but also correlates with impaired quality of life (10), decreased survival rates (11), and compromised treatment outcomes among individuals diagnosed with this disease (12). Evidence provided by a Meta-analysis also suggests that depression may have etiologic significance and prognostic implications in lung cancer, emphasizing the importance of early detection and effective intervention for depressive symptoms in lung cancer patients (13). However, identifying depression in individuals with lung cancer can be challenging due to its overlap with other manifestations such as sleep disturbances, fatigue, and loss of appetite.

The current therapeutic approach to managing depression in lung cancer is limited. Antidepressants are commonly utilized in clinical practice. The utilization of antidepressants in lung cancer patients has been shown to ameliorate depressive symptoms, shorten hospitalization duration (14), reduce resistance to tumor necrosis factor-related apoptosis-inducing ligands (15), and impede tumor growth (14). However, the precise mechanism underlying the effectiveness of antidepressants in patients with both lung cancer and depression remains elusive. Moreover, non-pharmacological interventions such as exercise training (16) and psychological therapies (17) have demonstrated efficacy in alleviating depressive symptoms among individuals diagnosed with lung cancer. However, a scarcity of robust evidence derived from rigorous scientific research persists.

The impact of depression on lung cancer patients extends beyond mental health, significantly influencing patients’ physiological status, treatment response, and overall quality of life (18, 19). Therefore, it is crucial to attain a comprehensive understanding of the current research landscape regarding depression in lung cancer patients, particularly concerning effective treatment strategies. This not only facilitates a clearer comprehension of the existing knowledge base but also identifies critical research gaps and delineates directions for future investigations. Ultimately, this endeavor aims to enhance patients’ quality of life and treatment adherence while pragmatically improving their treatment outcomes. Bibliometrics is a widely employed scientific methodology for investigating research progress (20), enabling researchers to swiftly evaluate scientific information and acquire dependable and practical knowledge in a specific research domain (21). However, there remains an insufficient amount of data pertaining to these specific aspects. Therefore, we employed CiteSpace software to conduct a bibliometric analysis of the association between depression and lung cancer, covering the period from 2014 to 2024. Our aim is to furnish researchers with innovative insights into the domains of depression in lung cancer, thereby establishing a scientific foundation for future research directions. This endeavor aims to facilitate the optimization of clinical treatments and enhance patient management strategies.

## 2 Methods

### 2.1 Data source and search strategies

Two independent authors conducted a search for relevant publications on the Web of Science (http://webofscience.com). The data was collected from the Web of Science Core Collection (WoSCC) database. WoSCC was selected as the data source due to its extensive disciplinary coverage and inclusion of high-impact literature, establishing it as one of the most authoritative and widely utilized databases in the field of bibliometrics. WoSCC not only encompasses high-quality journal articles but also offers detailed citation information, which is crucial for tracking research trends, analyzing scholarly impact, and identifying key literature within the discipline.

We utilized the search terms “lung,” “cancer,” and “depression” along with their relevant synonyms or abbreviations, followed by a comparison of respective findings to ensure the integrity and accuracy of our search results. The search query was TS= (lung cancer OR lung tumor OR lung tumour OR lung carcinoma OR lung neoplasm OR lung malignancy OR lung adenocarcinoma OR pulmonary cancer OR pulmonary tumor OR pulmonary tumour OR pulmonary carcinoma OR pulmonary neoplasm OR pulmonary malignancy OR pulmonary adenocarcinoma) AND TS= (depression OR depressive disorder OR depressive symptom OR emotional depression OR depress). The inclusion criteria of this study were as follows (1): studies published between January 1, 2014, and December 31, 2024 (2); studies that were an “article” or a “review article”, with no limitation in the language of the publication. The following exclusion criteria are established: 1) Exclusion of letters, meeting abstracts, meetings, proceeding papers, editorial materials, early access publications, and other non-article or article review literature; 2) Literature that is not pertinent to the research topic will also be excluded.

**Figure 1:**
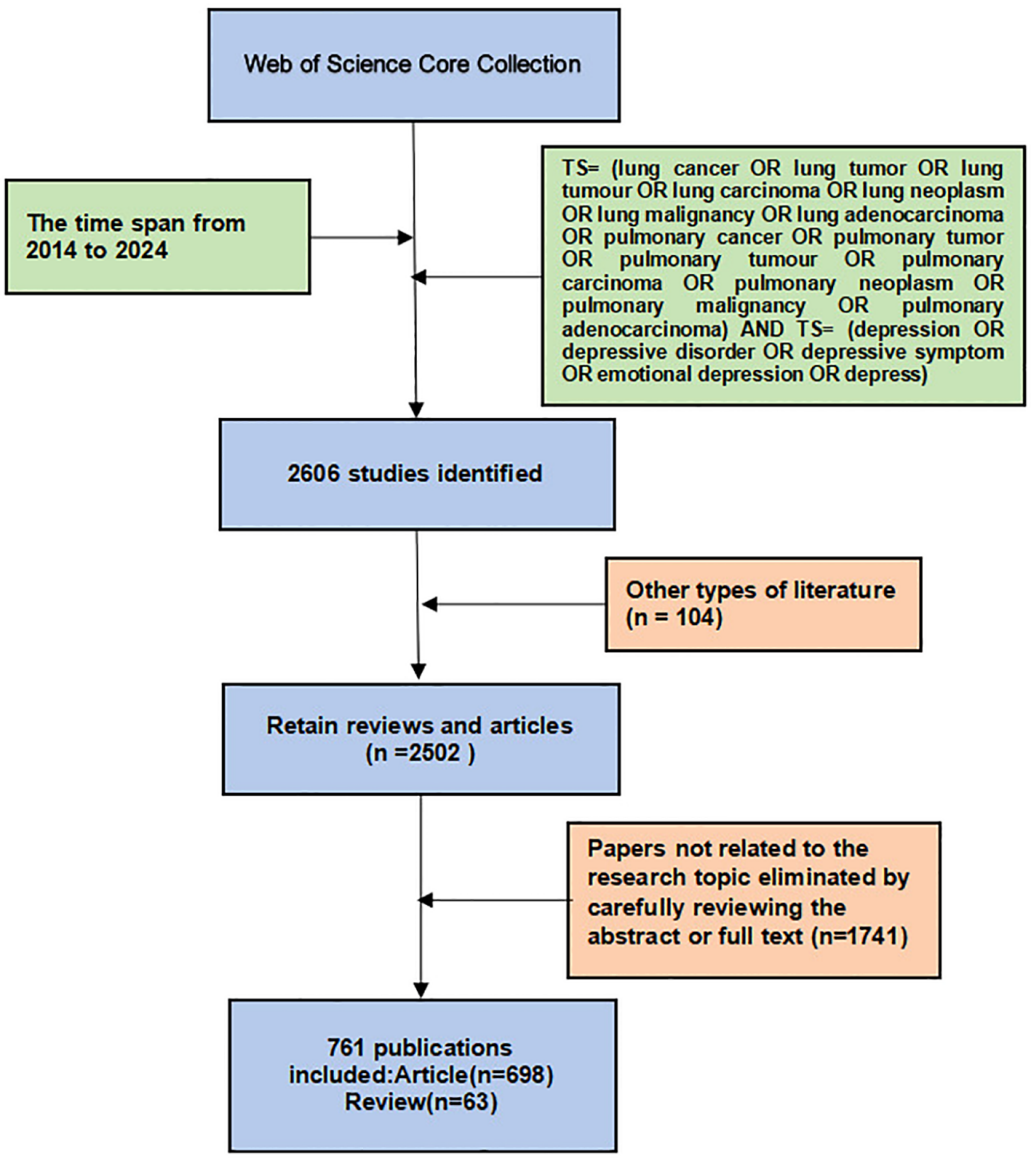
The implementation process of the study. (**Supplementary Material**).

All database searches were conducted on the same day (January 28, 2025) to ensure consistency. The screening process, however, was conducted over an extended period. The two authors (WL L and S C) independently screened the search results by reviewing titles and abstracts, and when necessary, reading the full text. Papers unrelated to depression in lung cancer were excluded. Any divergent opinions were resolved through consultation or, if needed, reviewed by an experienced corresponding author (CE W and F L). Ultimately, 761 articles that exclusively addressed the topic of lung cancer and depression were included. The specific publication screening flowchart is shown in **Figure 1**. All records and references were exported, saved as plain text files and stored as download_txt files.

### 2.2 Data analysis

CiteSpace (Version 6.2.R6) was regarded as an excellent visualization tool invented by Professor Chaomei Chen. We selected CiteSpace as our bibliometric analysis tool primarily due to its distinctive advantages in constructing scientific knowledge graphs and conducting visualization analyses. The fundamental principle of CiteSpace involves the construction of citation networks, which facilitates the examination of interrelationships among scholarly literature and the generation of knowledge graphs within a specific field. This process enables the identification of key research themes and emerging trends. Additionally, CiteSpace supports trend analysis based on time series data, effectively illustrating the dynamic changes in research progress over time (22, 23).

The parameters for CiteSpace were meticulously configured as follows: The time frame was set from 2014 to 2024 to encompass the entirety of the relevant data. Each node within the visualizations represents a distinct element, such as co-authors, institutions, countries, keywords, cited literature, cited authors, and cited journals. For the sake of consistency and comparability, all remaining parameters were kept at their default settings. The resulting data was imported into Microsoft Excel 2019 for graph generation, facilitating the creation of charts illustrating the annual publication volume and cumulative publication volume over time.

## 3 Results

### 3.1 Publication trends

**Figure 2:**
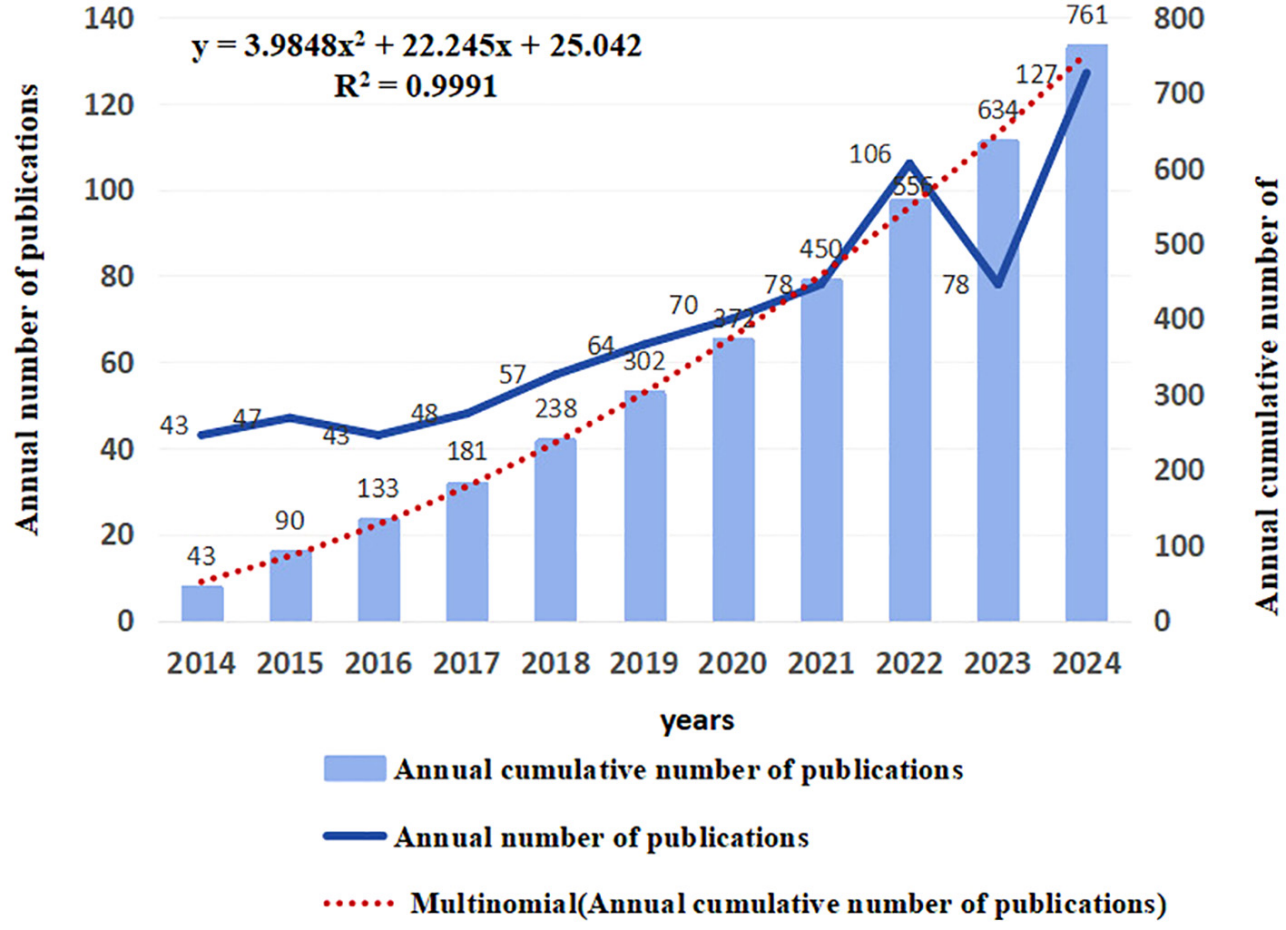
Annual and cumulative growth trend of publications.

The annual number of publications serves as a pivotal metric in assessing the progression of scientific inquiry, offering insights into the evolving landscape of knowledge within a given field. A total of 761 publications in the lung cancer and depression research field were identified, which were published between 2014 and 2024, and consisted of 698 original and 63 review articles. The trends in publication frequency are visually depicted in **Figure 2**, illustrating a fluctuating upward trajectory.

The number of annual articles increased steadily from 43 in 2014 to 127 in 2024, and the annual output peaked at 127 articles in 2024. Overall, researchers’ enthusiasm for lung cancer-related depression continues unabated. This analysis revealed a growth trend accurately expressed by the exponential function y = 3.9848x^2^ + 22.245x + 25.042 (R^2^ = 0.9991). In conclusion, the steady increase in the annual output of articles over the past 10 years indicates that depression in lung cancer is receiving increasing attention from researchers.

### 3.2 Geographical contributions

#### 3.2.1 International collaborations

**Figure 3:**
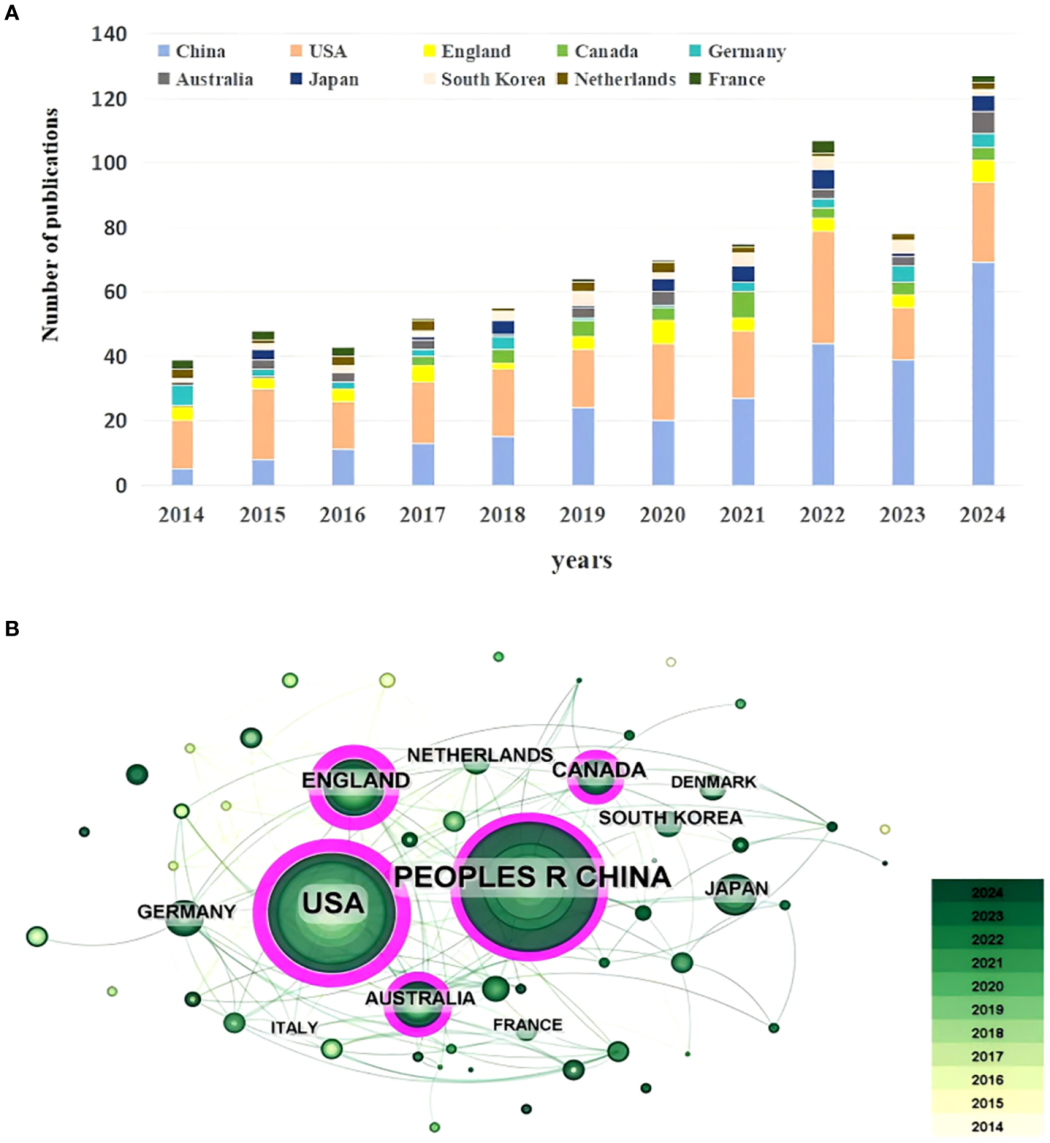
**(A)** The changing trend of the annual publication quantity in the top 10 countries. **(B)** The overlay visualization map of counties co-occurrence analysis.

Research on depression in lung cancer is a globally recognized subject, over the span of 2014-2024, contributions from 58 countries were observed in the selected publications. The top ten productive countries were ranked, as shown in **Table 1**. **Figure 3A** illustrates the changes in publication output for the top 10 countries from 2014 to 2024. In terms of the number of publications, China remained the most productive (275, 36.14%), followed by the USA (231, 30.35%), and England (48, 6.31%). The greater the centrality, the stronger the partnership. The top three countries in terms of centrality were the USA (0.43), England (0.41), and Australia (0.19). Despite holding the position of the country with the highest number of publications, China exhibits a significantly lower level of centrality compared to the USA(0.43), which ranks second, and England(0.41), which ranks third. This indicates that both the USA and England possess a relatively strong influence in the field of lung cancer-related depression, which could potentially motivate China to publish articles of higher quality.

**Table 1:** The top 10 productive countries.

Rank	Country	Count	Percentage (%, of 761)	Centrality
1	China	275	36.14	0.12
2	USA	231	30.35	0.43
3	England	48	6.31	0.41
4	Canada	37	4.86	0.16
5	Germany	33	4.34	0.09
6	Australia	31	4.07	0.19
7	Japan	30	3.94	0
8	South Korea	30	3.94	0
9	Netherlands	24	3.15	0.09
10	France	19	2.50	0.01

“Centrality” denotes the academic influence and network connectivity of a country within the research domain, a higher Centrality value signifies that the country’s research activities and impact in the field are more pronounced.

As indicated in **Figure 3B**, each node in the figure represents a country, and the size of the node indicates the publication output of the country. Different colors within a node represent distinct time intervals; the darker the hue, the more recent the published article is in relation to the present. The lines between the nodes denote cooperation between countries, and the thicker the lines are, the closer the entities’ cooperation. Among the top 10 productive countries, robust collaboration was observed, serving as the cornerstone of the international collaborative landscape. China, the USA, England, Canada, and Australia exhibited a centrality value greater than 0.1 among these leading countries, indicating their pivotal roles as active centers for collaboration. Overall, international collaboration in this research domain is constrained and lacks effective connectivity. Despite China’s substantial publication output, its level of international collaboration remains comparatively lower. It is recommended that countries enhance their academic cooperation with other nations to foster greater scholarly exchange and global research partnerships.

#### 3.2.2 Institutional collaborations

A total of 305 institutions contributed to research on depression in lung cancer from 2014 to 2024. The top ten productive institutions were ranked, as shown in **Table 2**. Harvard University (58, 7.62%) emerges as the most productive institution, followed by Massachusetts General Hospital (35, 4.60%), and Harvard Medical School (31, 4.07%). Among the top 10 institutions, Among the top 10 institutions, one is located in Canada, and one is in China, while the remaining ones are headquartered in the USA. Notably, only one of the top 10 institutions in terms of publication volume is affiliated with Chinese institutions, despite China being the most productive country. This observation suggests that despite the presence of numerous institutions in China publishing articles on depression in lung cancer, there remains a relatively low quantity of published articles by these institutions. The top three institutions in terms of centrality were Harvard University (0.21), University of Toronto (0.15), and Memorial Sloan Kettering Cancer Center (0.09). Among all institutions, only Harvard University and the University of Toronto demonstrated Centrality values exceeding 0.1. This underscores the substantial impact and authority of these two institutions on scholars engaged in depression in lung cancer research.

**Table 2:** The top 10 productive institutions.

Rank	Institution	Count	Percentage(%, of 761)	Centrality	Country
1	Harvard University	58	7.62	0.21	USA
2	Massachusetts General Hospital	35	4.60	0.02	USA
3	Harvard Medical School	31	4.07	0.02	USA
4	Memorial Sloan Kettering Cancer Center	23	3.02	0.09	USA
5	University of Toronto	21	2.76	0.15	Canada
6	University of California System	21	2.76	0.03	USA
7	Dana-Farber Cancer Institute	20	2.63	0.02	USA
8	Veterans Health Administration	19	2.50	0.03	USA
9	US Department of Veterans Affairs	19	2.50	0.03	USA
10	Central South University	17	2.23	0.06	China

**Figure 4:**
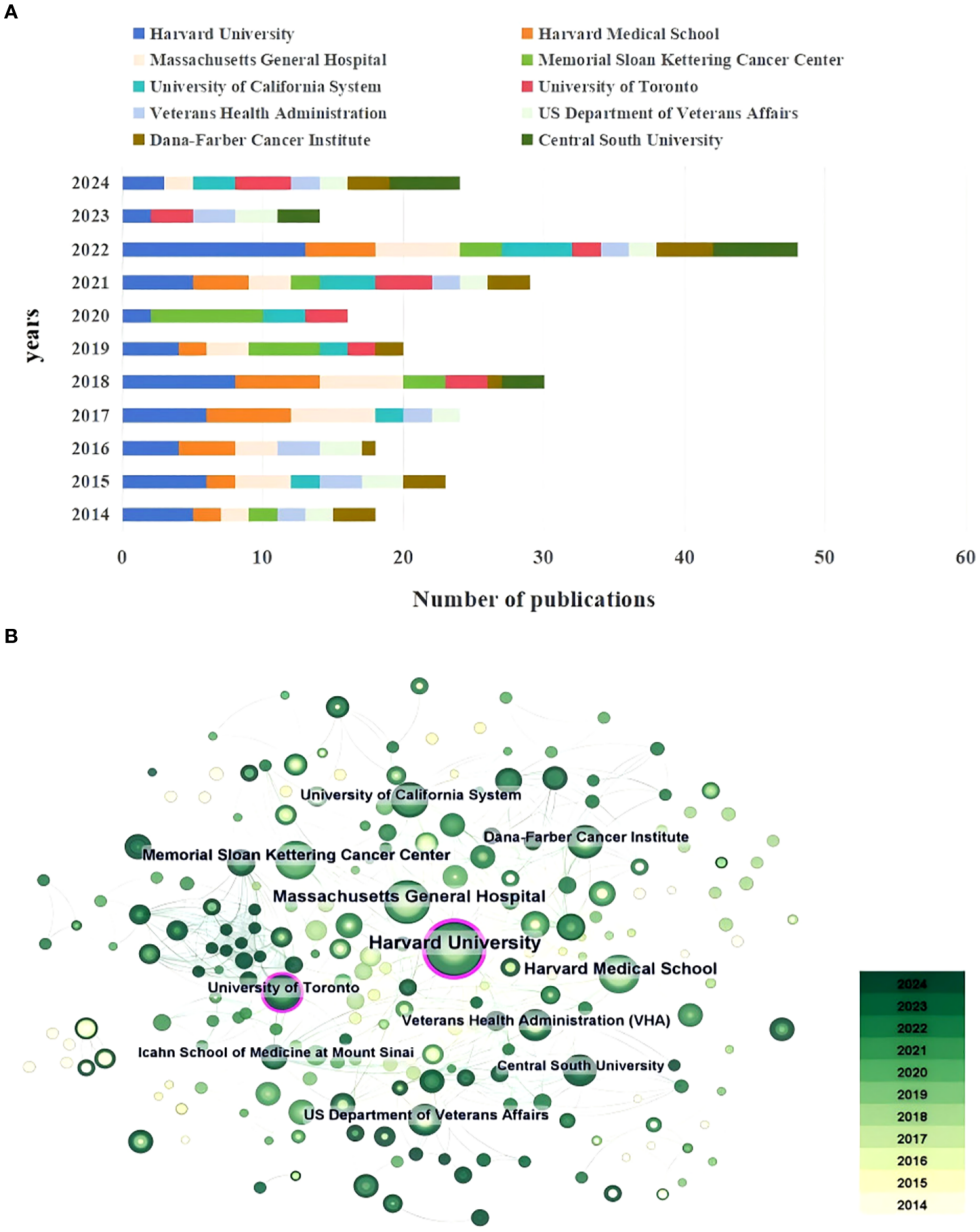
**(A)** The changing trend of the annual publication quantity in the top 10 institutions. **(B)** The overlay visualization map of institutions co-occurrence analysis.

The cumulative annual publications of the top 10 institutions are shown in **Figure 4A**. We also examined the institutions’ co-authorship (**Figure 4B**). Citespace was used to build a co-authorship network map of institutions to study cooperation among institutions. As indicated in **Figure 4B**, each node in the figure represents an institution, and the size of the node indicates the publication output of the institution. Different colors inside the nodes signify different time intervals. The lines between the nodes denote cooperation between institutions, and the thicker the lines are, the closer the entities’ cooperation. Different colors within a node represent distinct time intervals; the darker the hue, the more recent the published article is in relation to the present. The top 3 productive institutions included Harvard University (58, 7.62%), Massachusetts General Hospital (35, 4.60%), and Harvard Medical School (31, 4.07%), which form the core of the complex cooperative network. Harvard University (0.21) has the highest number of collaborative partners, followed by the University of Toronto (0.15); these two institutions are at the forefront of this field of research. The institutions with the highest number of publications mainly consist of universities, hospitals, and medical centers. Despite the presence of some degree of collaboration among institutions, its extent remains limited. In addition, **Figure 4B** illustrates that several institutions are represented by darker nodes, indicating that a number of these institutions have recently initiated research in the area of lung cancer-related depression. In conjunction with **Figure 2**, the annual publication count demonstrates a consistent upward trajectory, indicating an escalating level of scholarly attention devoted to this particular domain.

### 3.3 Authors/co-cited authors analysis

#### 3.3.1 Authors collaborations

**Figure 5:**
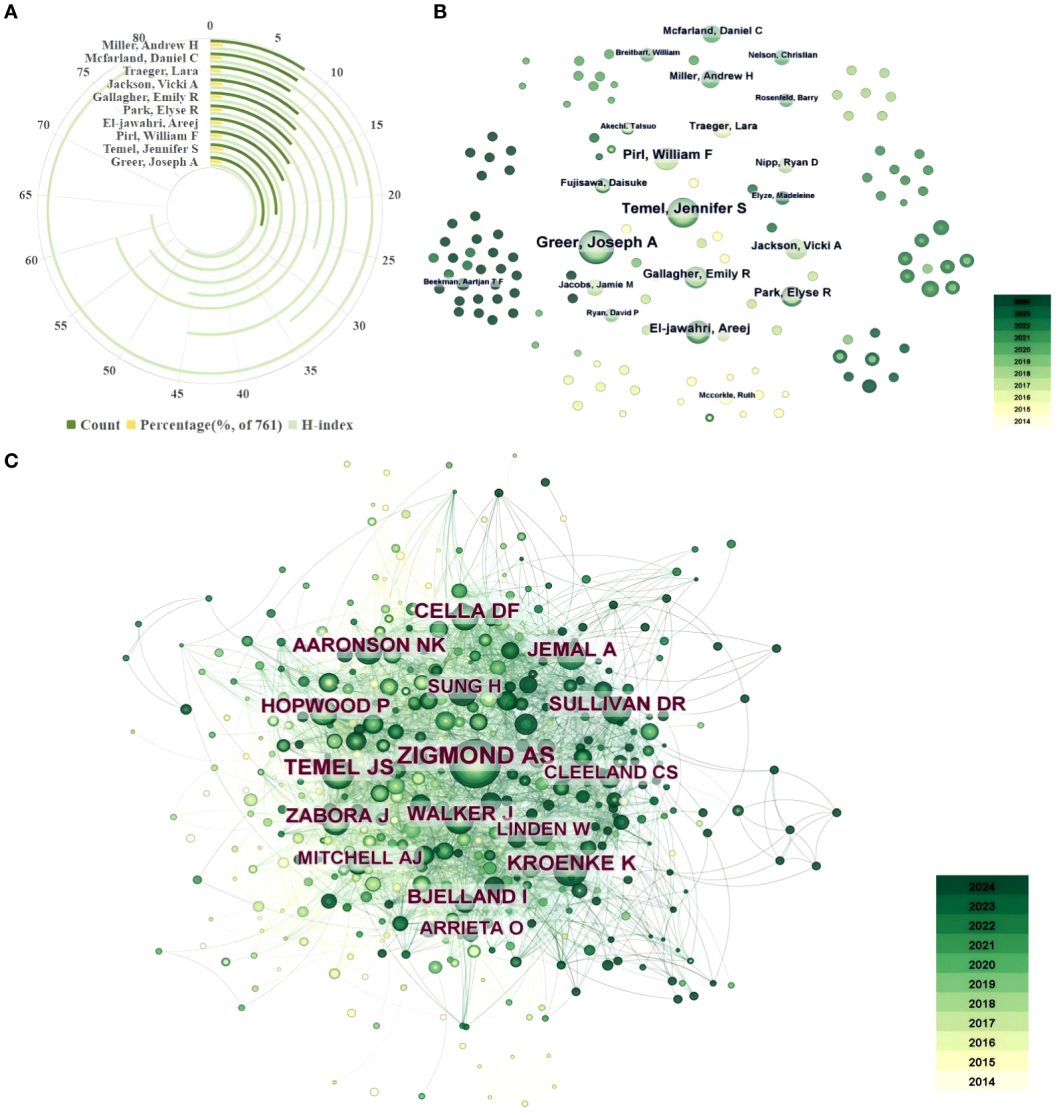
**(A)** The H-index and count percentage of the top 10 productive authors. **(B)** The overlay visualization map of authors co-occurrence analysis. **(C)** The overlay visualization map of co-cited authors co-occurrence analysis.

A total of 4,667 authors contributed to published articles on lung cancer and depression. **Table 3** and **Figure 5A** show the top 10 most productive authors with the number of publications, Centrality, H-index, et al. Notably, the H-index data for these authors was obtained from the Web of Science database, with a search date of January 30, 2025. Greer, Joseph A (25, 3.29%, H-index50), Temel, Jennifer S (22, 2.89%, H-index63), and Pirl, William F (16, 2.10%, H-index56) were the most productive contributors to this field, while Miller, Andrew H had the highest H-index of 78. Notably, all these leading authors are based in the USA, reflecting the country’s significant contributions to the field. Despite China being the country with the highest number of articles, none of the top 10 published authors are affiliated with Chinese institutions. This observation implies that while there is a substantial presence of Chinese authors contributing to research on lung cancer-related depression, their individual publication output remains relatively modest, thus limiting their global influence.

**Table 3:** The top 10 productive authors.

Rank	Author	Count	Percentage(%, of 761)	Centrality	H-index	Country
1	Greer, Joseph A	25	3.29	0	50	USA
2	Temel, Jennifer S	22	2.89	0	63	USA
3	Pirl, William F	16	2.10	0.01	56	USA
4	El-jawahri, Areej	14	1.84	0	46	USA
5	Park, Elyse R	12	1.58	0	60	USA
6	Gallagher, Emily R	11	1.45	0	26	USA
7	Jackson, Vicki A	10	1.31	0	45	USA
8	Traeger, Lara	8	1.05	0	34	USA
9	Mcfarland, Daniel C	8	1.05	0	19	USA
10	Miller, Andrew H	8	1.05	0	78	USA

**Figure 5B** is a co-authorship analysis visualization generated by citespace. Each node represents an author, and the lines between the nodes indicate the connections between authors. The yellow color indicates that the author has previously focused on the research area, while the intensity of the green color reflects the recency of the author’s publications. The greener the node, the more recent the publication of the author’s paper. In the field of depression in lung cancer, several research teams have been established. Among them, the team led by Greer, Joseph A., Temel, and Jennifer S. has demonstrated the highest productivity in terms of published articles and the number of authors involved. Additionally, all top 10 authors in terms of article publications are affiliated with this particular team. Therefore, it can be inferred that this team constitutes the central nucleus within this specific domain of research. Unfortunately, the research clusters are generally dispersed, irrespective of whether the team led by Greer, Joseph A. and Temel, Jennifer S, or other research groups, intra-team collaboration is highly cohesive while inter-team collaboration remains scarce. This observation implies a limited extent of international cooperation in the field of depression in lung cancer.

#### 3.3.2 Co-cited authors analysis

The citation count serves as a quantitative measure of the scientific impact. **Table 4** presents the top 10 authors based on co-citations. Notably, the country for these authors was obtained from the Web of Science database, with a search date of January 30, 2025. A S Zigmond (185 citations) ranked first, followed by Jennifer S Temel (101 citations), and Kroenke Kurt (80 citations). The co-cited authors predominantly originate from the United States, alongside contributors from England, and the Netherlands, highlighting the global nature and collaborative spirit of the research. It is noteworthy that among the top 10 highly cited authors, only Temel Jennifer S from the United States possesses a relatively substantial publication record. Temel Jennifer S features in the list of the top 10 most prolific authors with a total of 22 publications (the second highest). This demonstrates his prolific publication record, characterized by a substantial volume of high-quality articles that have garnered widespread recognition within the academic community.

**Table 4:** The top 10 co−cited authors.

Rank	Cited author	Count	Centrality	Country
1	Zigmond, A.S	185	0.06	England
2	Temel, Jennifer S	101	0.06	USA
3	Kroenke, Kurt	80	0.06	USA
4	Cella, D F	78	0.07	USA
5	Jemal, Ahmedin	70	0.03	USA
6	Aaronson, N K	70	0.07	The Netherlands
7	Hopwood, P	63	0.06	England
8	Sullivan DR	61	0.05	USA
9	Zabora, J	59	0.04	USA
10	Walker, Jane	56	0.08	England

**Figure 5C**. is a co-cited authors analysis visualization generated by citespace. The circle size corresponds to the number of citations received by an author’s paper. The lines connecting the circles indicate the co-citation relationship between the two authors. The intensity of the node’s color correlates with its temporal proximity to the present; darker hues indicate a closer reference to current time. The largest node in the figure is Zigmond, A.S, followed by Temel, Jennifer S, indicating that their articles have a certain influence in the field of lung cancer in depression. Of the larger nodes, the centers exhibited a yellowish hue, while the outer edges displayed a dark green coloration. A number of small nodes are presented in dark green, indicating that the article on lung cancer-related depression has garnered increased scholarly attention in recent years.

### 3.4 Journals/cited journals analysis

#### 3.4.1 Journals analysis

**Figure 6:**
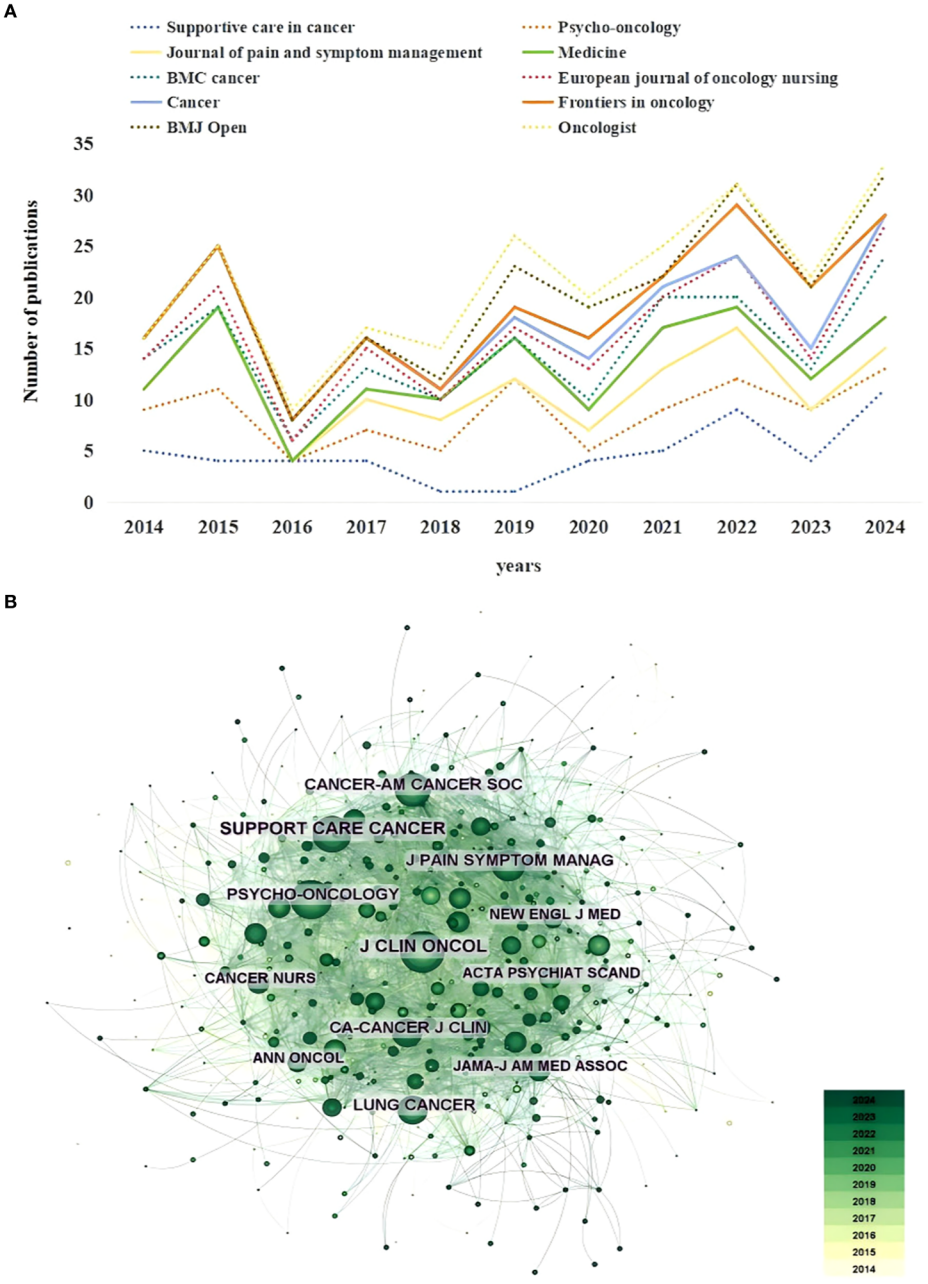
**(A)** The changing trend of the annual publication quantity in the top 10 journals. **(B)** The overlay visualization map of co-cited journals co-occurrence analysis.

A total of 249 academic journals have published articles on depression in lung cancer. **Table 5** summarizes the top 10 journals, incorporating publication volume, Impact Factors (IF), and Journal Citation Reports (JCR) category to comprehensively assess the influence of journals. The volume of publications serves as an indicator of a journal’s attention and activity within the field, to some extent reflecting the research frontiers and development trends in the domain. Supportive care in cancer (52 publications, 6.83%, IF=2.8, Q3) has the highest output in depression of lung cancer, followed by Psycho-oncology (44 publications, 5.78%, IF=3.3, Q3) and Journal of pain and symptom management (29 publications, 3.81%, IF=3.2, Q1). Journal of Cancer (15 publications, 1.97%, IF=6.1, Q1) had the highest IF among the 10 most productive journals. The top 10 journals in terms of the number of articles published account for 31.41% of the total publications. The IF of these journals, as reported in JCR 2023, range from 1.3 to 6.1. Furthermore, 70% of the journals belong to Q2 or Q3, indicating their influential status. Interestingly, the 10 most prolific journals are predominantly focused on oncology and originate exclusively from developed countries: five from England, three from the USA, one from Germany, and one from Switzerland. In addition, the annual publication volume of the top 10 journals was analyzed to better understand their annual publication trends (**Figure 6A**). It is noteworthy that Supportive care in cancer reached its peak in 2024 with a total of 11 articles, making it the most prolific among the 10 journals.

**Table 5:** The top 10 most prolific journals.

Rank	Journal	Country	IF 2023	5-years IF	JCR Category	JCR	Open access	Count	Percentage (%, of 761)
1	Supportive care in cancer	Germany	2.8	3.2	ONCOLOGY	Q1	YES	52	6.83
2	Psycho-oncology	England	3.3	4.4	ONCOLOGY/PSYCHOLOGY	Q2/Q1	NO	44	5.78
3	Journal of pain and symptom management	USA	3.2	3.8	CLINICAL NEUROLOGY	Q2	NO	29	3.81
4	Medicine	USA	1.3	1.6	MEDICINE, GENERAL & INTERNAL	Q2	NO	21	2.76
5	BMC cancer	England	3.4	3.8	ONCOLOGY	Q2	NO	19	2.50
6	European journal of oncology nursing	England	2.7	3	ONCOLOGY	Q3	NO	16	2.10
7	Cancer	USA	6.1	6.7	ONCOLOGY	Q1	YES	15	1.97
8	Frontiers in oncology	Switzerland	3.5	4	ONCOLOGY	Q2	YES	15	1.97
9	BMJ Open	England	2.4	2.7	MEDICINE, GENERAL & INTERNAL	Q1	YES	14	1.84
10	Oncologist	England	4.8	5.3	ONCOLOGY	Q1	YES	14	1.84

#### 3.4.2 Cited journals analysis

In this study, a total of 520 co-cited journals were identified. Co-citation analysis is used to evaluate the degree of relationship between articles, exploring their development and evolution. Citations are one of the main factors in determining a journal’s academic impact. **Table 6** summarizes the top 10 co-cited journals, total citation count, IF) and JCR category to comprehensively assess the influence of journals. Journal of Clinical Oncology (cited 480 times, IF42.1, Q1) has the highest co-citation frequency, followed by Supportive Care in Cancer (cited 421 times, IF2.8, Q3), Psycho-Oncology (cited 390 times, IF3.3, Q3). As shown in **Table 6**, except for Supportive Care in Cancer and Psycho-Oncology all the top 10 co-cited journals are either Q1, Q2 or Q3, with 70% of co-cited journals belonging to Q1. The average IF of the top 10 co-cited journals is 69.02.

**Table 6:** Top 10 co-citation journals.

Rank	Cited journals	Country	IF 2023	JCR	Citations	Centrality
1	Journal of Clinical Oncology	USA	42.1	Q1	480	0.01
2	Supportive Care in Cancer	Germany	2.8	Q3	421	0.01
3	Psycho-Oncology	England	3.3	Q3	390	0.02
4	Cancer	USA	6.1	Q1	354	0.01
5	Journal of Pain and Symptom Management	USA	3.2	Q1	330	0.01
6	Lung Cancer	Ireland	4.5	Q2	263	0.02
7	CA: A Cancer Journal for Clinicians	USA	503.1	Q1	245	0.02
8	Acta Psychiatrica Scandinavica	USA	5.3	Q1	202	0
9	JAMA-Journal of the American Medical Association	USA	63.1	Q1	201	0.02
10	Annals of Oncology	England	56.7	Q1	199	0.02

An analysis of **Tables 5** and **6** reveals that Supportive care in cancer ranked first in terms of the number of published articles and second in terms of citations; Psycho-oncology ranked second in terms of the number of published articles and third in terms of citations; Journal of Pain and Symptom Management ranked third in terms of the number of published articles and fifth in terms of citations; Cancer ranked seventh in terms of the number of published articles but fourth in terms of citations. This implies that these journals exert a significant influence in the domain of depression in lung cancer.

The analysis was carried out using the visualization software citespace and the results are shown in **Figure 6B**. The larger the node, the greater the number of citations associated with this journal. A deeper color indicates that the citations are more recent. Journal of Clinical Oncology could be considered the most influential journal in the field of lung cancer-related depression. The majority of these journals pertain to cancer research, while a subset focuses on psychological studies.

### 3.5 Co-cited articles analysis

In total of the 538 co-cited references retrieved, the top 10 co-cited references are shown in **Table 7**. Highly co-cited references are frequently cited together by other articles, thus establishing them as a fundamental knowledge base within a specific academic domain. The citation volume of the top 10 co-cited references in this study ranged from 12 to 49. The ten articles were all cancer-related, with six directly addressing lung cancer and two focusing on emotional issues in patients with lung cancer. These 10 references can be categorized into four types: Epidemiology of cancer, assessment of depressive symptoms in lung cancer patients, impact of some treatments on lung cancer patients, and impact of depressive symptoms on survival in lung cancer patients. The top three citations are as follows: In 2021, Sung H et al. published a paper titled “Global Cancer Statistics 2020: GLOBOCAN Estimates of Incidence and Mortality Worldwide for 36 Cancers in 185 Countries” in the Journal of CA: a cancer journal for clinicians (24); Wang YH et al., 2020, published a paper titled “Depression and anxiety in relation to cancer incidence and mortality: a systematic review and meta-analysis of cohort studies” in the Journal of Molecular psychiatry (13); Sullivan DR et al., 2016, published a paper titled “Longitudinal Changes in Depression Symptoms and Survival Among Patients With Lung Cancer: A National Cohort Assessment” in the Journal of clinical oncology (25).

**Table 7:** The top 10 co-cited references.

Rank	First author	Year	Title	Citations	Centrality	Journal
1	Sung H	2021	Global Cancer Statistics 2020: GLOBOCAN Estimates of Incidence and Mortality Worldwide for 36 Cancers in 185 Countries	49	0.03	CA: a cancer journal for clinicians
2	Wang YH	2020	Depression and anxiety in relation to cancer incidence and mortality: a systematic review and meta-analysis of cohort studies	30	0.14	Molecular psychiatry
3	Sullivan DR	2016	Longitudinal Changes in Depression Symptoms and Survival Among Patients With Lung Cancer: A National Cohort Assessment	20	0.03	Journal of clinical oncology
4	Morrison EJ	2017	Emotional Problems, Quality of Life, and Symptom Burden in Patients With Lung Cancer	20	0.08	Clinical lung cancer
5	Bade BC	2020	Lung Cancer 2020: Epidemiology, Etiology, and Prevention	18	0.03	Clinics in chest medicine
6	Yan XR	2019	Prevalence and risk factors of anxiety and depression in Chinese patients with lung cancer: a cross-sectional study	17	0.18	Cancer management and research
7	Temel JS	2017	Effects of Early Integrated Palliative Care in Patients With Lung and GI Cancer: A Randomized Clinical Trial	15	0.07	Journal of clinical oncology
8	Temel JS	2010	Early palliative care for patients with metastatic non-small-cell lung cancer	13	0.13	The New England journal of medicine
9	Peddle-McIntyre CJ	2019	Exercise training for advanced lung cancer	13	0.11	The Cochrane database of systematic reviews
10	Chen WQ	2016	Cancer statistics in China, 2015	12	0.05	CA: a cancer journal for clinicians

**Figure 7:**
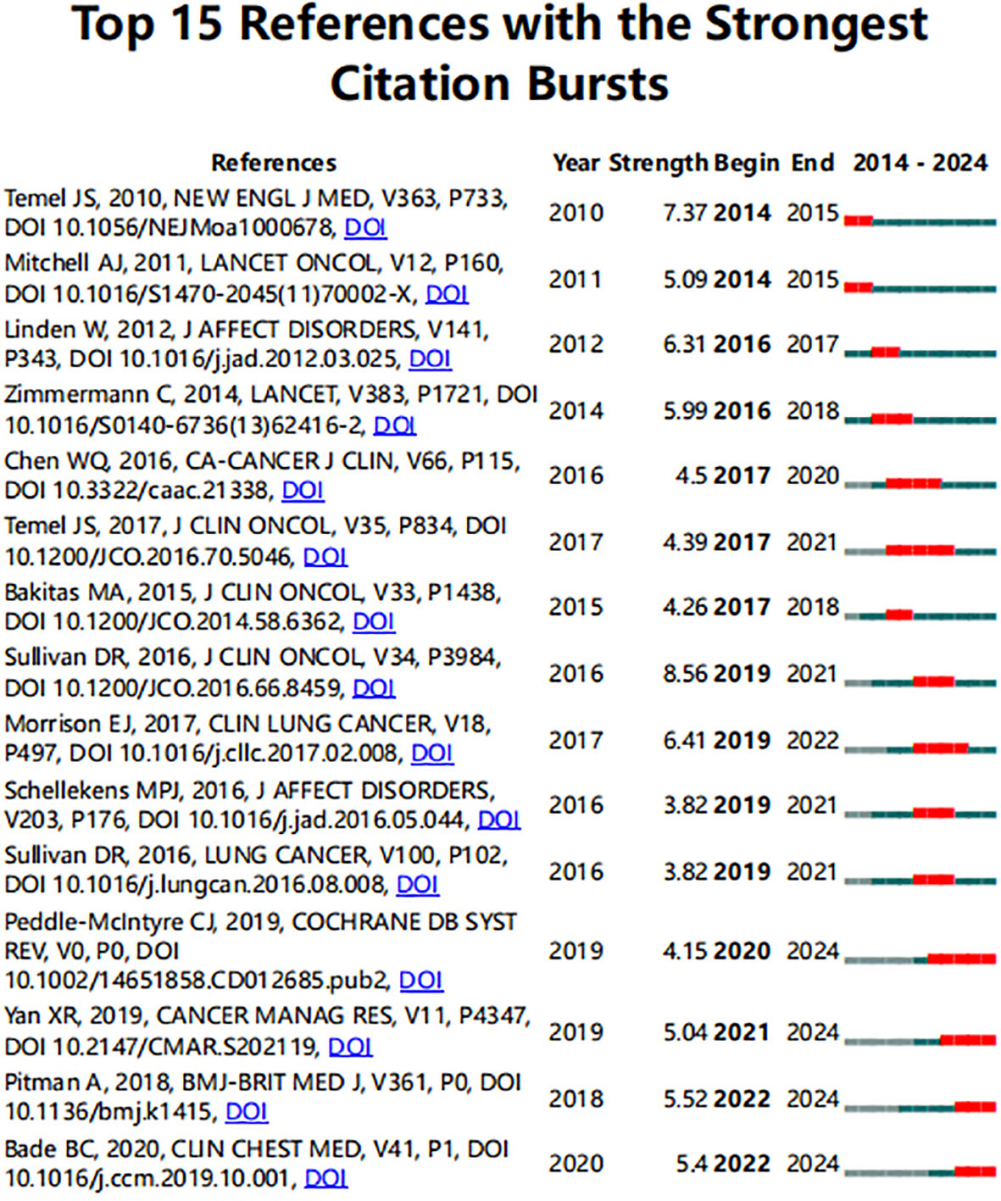
Top 15 references with the strongest citation bursts. (**Supplementary Material**).

Citation bursts, characterized as frequent citations at a given point in time, can be used to identify the evolution of a knowledge domain. Our analysis identified the top 15 references with the most significant citation bursts (**Figure 7**). Referencing bursts were displayed as a red line segment. A greater strength indicates a higher citation frequency. There has been a significant increase in references experiencing a burst in citations after 2016, indicating rapid development in the field of depression in lung cancer since 2016. The paper “Longitudinal Changes in Depression Symptoms and Survival Among Patients With Lung Cancer: A National Cohort Assessment” published in Journal of clinical oncology (2023IF42.1, Q1) by Sullivan DR exhibited the strongest citation burst (strength = 8.56, from 2019 to 2021) among these references, with bursts occurring from 2019 to 2021 (25), followed by “Early palliative care for patients with metastatic non-small-cell lung cancer” published in The New England journal of medicine (2023IF96.2, Q1) by Jennifer S Temel et al. with a citation burst lasting from 2014 to 2015 (strength =7.37, from 2014 to 2015) (26). The study titled “Effects of Early Integrated Palliative Care in Patients With Lung and GI Cancer: A Randomized Clinical Trial” (Strength =4.39, from 2017 to 2021) by Jennifer S Temel et al., published in journal of clinical oncology (27), and the study titled “Exercise training for advanced lung cancer” (Strength =4.15, from 2020 to at least 2024) by Carolyn J Peddle-McIntyre et al., published in The Cochrane database of systematic reviews (28), were the longest duration of citation bursts. Overall, the burst strength of the top 15 references ranged from 3.82 to 8.56, while the most frequent burst duration was 5 years. These 15 references encompass three primary themes: the epidemiology pertaining to lung cancer-related depression, the detrimental effects of depression on individuals diagnosed with lung cancer, and the treatment for lung cancer patients, including palliative care and exercise therapy.

### 3.6 Keywords analysis

#### 3.6.1 Keyword co-occurrence

Keyword analysis offers a comprehensive overview of research trends that epitomize the journal, reflecting the focal point of an article or an individual author. The total sum of keywords in 761 documents is 379, with 75 keywords appearing more than 10 times. The top 20 keywords for frequency are listed in **Table 8**, the year corresponding to each keyword is the earliest year it occurred. Among them, the most common occurrence was “quality of life” (376 times), followed by “lung cancer” (313 times), and “depression” (192 times), which was consistent with the study theme. The top 20 keywords encompass various aspects of lung cancer, including its clinical manifestations, treatment modalities, patient management, and the psychological well-being of patients. Among the remaining keywords, it suggests a potential comorbidity of anxiety, pain, and other negative affective states in patients with depression related to lung cancer. It is worth noting that the term “quality of life” demonstrates the highest frequency, indicating the significance attributed by researchers and clinicians to assessing the quality of life among patients suffering from depression associated with lung cancer.

**Table 8:** Top 20 co-occurrence keywords involved.

Rank	Keyword	Frequency	Centrality	Year	Rank	Keyword	Frequency	Centrality	Year
1	quality of life	376	0.01	2014	11	symptoms	65	0.07	2014
2	lung cancer	313	0.01	2014	12	survival	65	0.03	2014
3	depression	192	0.02	2014	13	health	58	0.02	2014
4	prevalence	142	0.03	2014	14	management	56	0.04	2014
5	anxiety	121	0.04	2014	15	outcm	56	0.05	2014
6	breast cancer	84	0.06	2014	16	chemotherapy	55	0.1	2014
7	distress	81	0.03	2014	17	fatigue	54	0.05	2014
8	palliative care	79	0.04	2014	18	survivors	54	0.07	2014
9	hospital anxiety	74	0.03	2014	19	validation	54	0.04	2014
10	psychological distress	66	0.06	2014	20	care	52	0.05	2015

**Figure 8:**
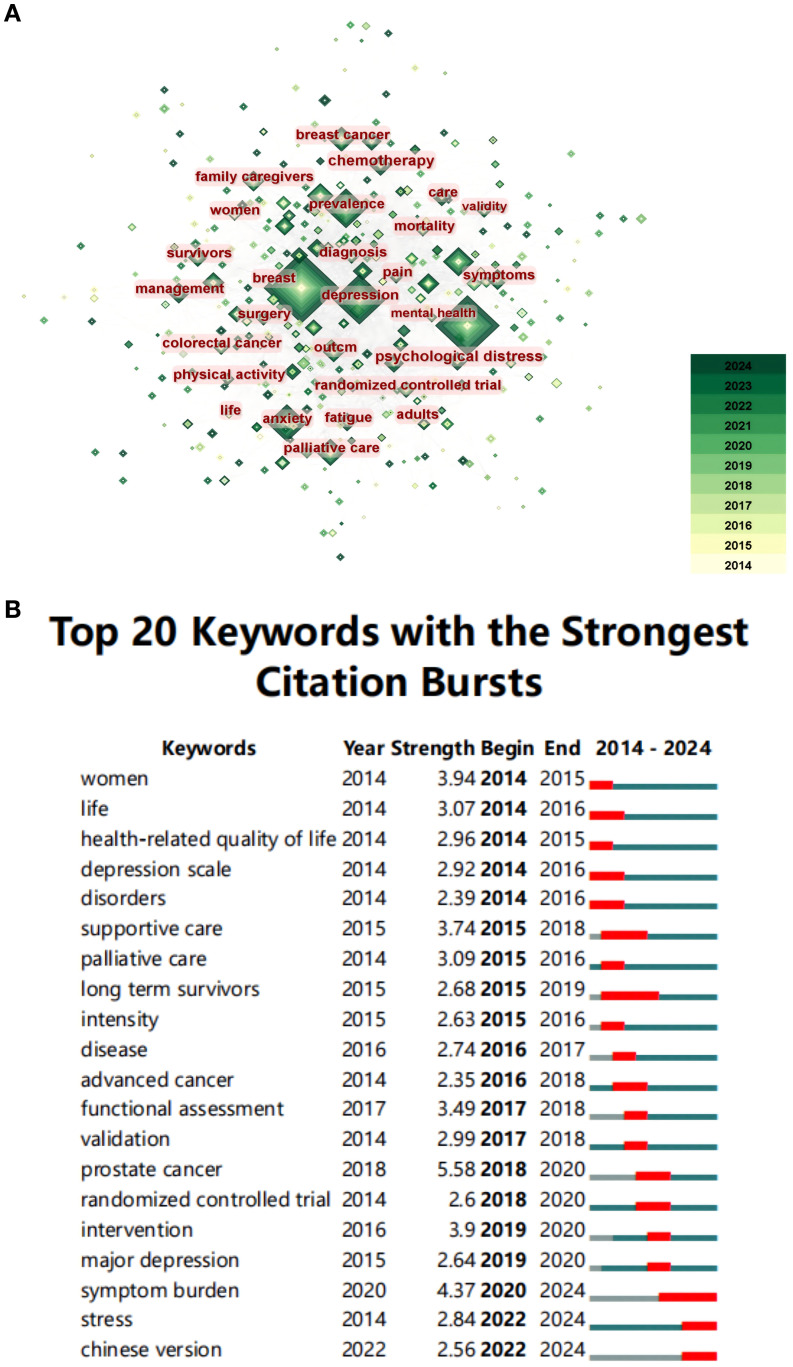
**(A)** The overlay visualization map of keywords co-occurrence analysis. **(B)** Top 20 keywords with the strongest citation bursts.

A co-occurrence knowledge map of keywords was generated using CiteSpace software (**Figure 8A**). The co-occurrence of keywords is the appearance of two keywords in the same publication. In the visualization map, the size of nodes indicates the frequency of occurrences of the keywords. The lines between the nodes represent their co-occurrence in the same publication. The deeper the color of the node, the closer the occurrence time of the highlighted keyword is to the present. The term “depression” emerged as a central node in the co-occurrence network, indicating their frequent mention in the literature. Surrounding the central nodes are significant terms like “prevalence”, “anxiety”, “breast cancer”, “mental health”, and “palliative care”, highlighting these as foundational and recurrent topics in the field. In conjunction with the duration of the nodes, this indicates that the epidemiology and management of depression related to lung cancer remain a persistent concern within this research domain.

#### 3.6.2 Keyword burst

CiteSpace was employed to analyze the keyword burst on depression in lung cancer from 2014 to 2024. **Figure 8B** illustrates the top 20 keywords with the highest burst intensity. Keyword burst analysis reflects the degree of acceptance and dissemination of major research topics. In the figure, the year represents the earliest year in which the keyword appears. Begin and end represent the times when the burst starts and ends, respectively. The blue line represents the time interval, and the red line represents the duration of the burst. Keywords with the strongest burst intensity include “prostate cancer” (Strength = 5.58, from 2018 to 2020), “symptom burden” (Strength = 4.37, from 2020 to at least 2024), and “women” (Strength = 3.94, from 2014 to 2015), indicating the widespread acceptance of these topics. Recent keywords in a burst state include “symptom burden” (Strength = 4.37, from 2020 to at least 2024), “stress” (Strength =2.84, from 2022 to at least 2024), and “Chinese version” (Strength =2.56, from 2022 to at least 2024), indicating the current hot spots in these areas. The terms “long term survivors” (Strength =2.68, from 2015 to 2019) and “symptom burden” (Strength =4.37, from 2020 to at least 2024) have demonstrated remarkable longevity, while the concepts of “symptom burden” (Strength =4.37, from 2020 to at least 2024), “stress” (Strength =2.84, from 2022 to at least 2024), “Chinese version” (Strength =2.56, from 2022 to at least 2024) have almost consistently maintained their prominence since the onset of the outbreak, indicating their enduring significance in future research endeavors.

#### 3.6.3 Keyword clustering and timeline

**Figure 9:**
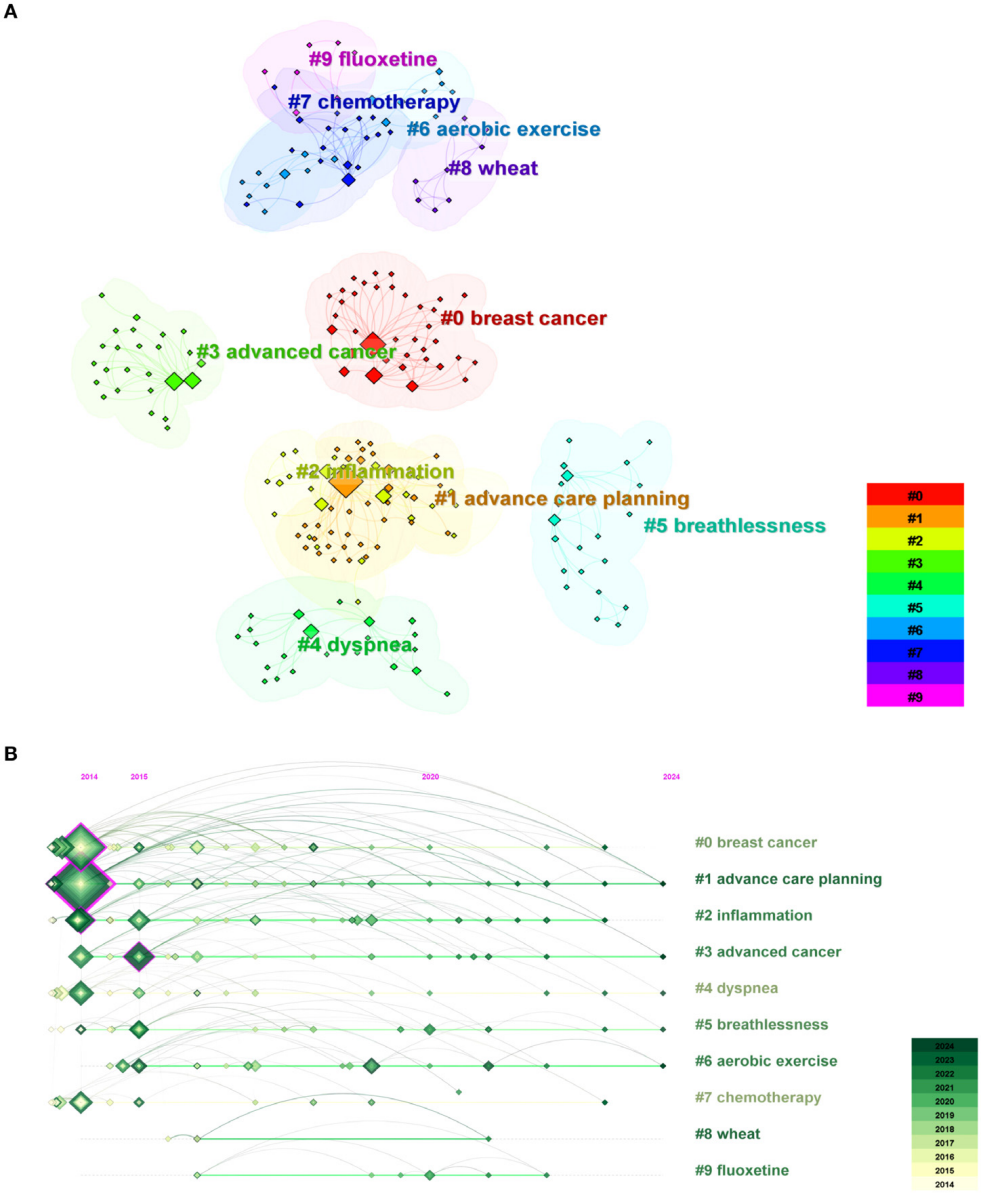
**(A)** The cluster view map of keywords analysis. **(B)** The cluster timeline view map of keywords analysis.

We utilized CiteSpace software to obtain the clustering function, selecting the top 50 items by usage since 2013 shown in **Figure 9A** (Q=0.5951, S=0.9388). In the keyword clustering analysis figure, a higher Modularity value (Q value) indicates a clearer delineation of subgroups within the network and suggests a more rational modular division. Conversely, the closer the Average silhouette value (S value) is to 1, the better the clustering effect; this reflects greater cohesion within clusters and enhanced differentiation between them. The Q and S values presented in **Figure 8** imply that the clustering depicted in this image is indeed reasonable. The size of the node positively correlates with the frequency of keyword occurrences, while the link between the two nodes represents their connection. The different colors represent distinct clusters, with a total of 10 clusters, numbered from 0 to 9.

CiteSpace was used to analyze the timeline view of depression in lung cancer from 2014 to 2024 (**Figure 9B**). The keyword timeline view describes the trend of changing research hotspots over time in a field. All keywords were categorized into 10 clusters, with the number of keywords in a cluster indicating the significance of that topic within the field. Analyzing the temporal dynamics of each cluster enables a more comprehensive exploration of the principal research themes in this domain from a microscopic perspective. Additionally, the distance from left to right for each cluster represents the start and end times of each cluster, while the size of the color-loaded points indicates the frequency of occurrence of the cluster’s label terms. Moreover, the colored lines depict co-occurrence relationships among distinct label terms in clusters.

According to **Figure 9A**, cluster #0 labeled “ breast cancer” was the largest cluster, followed by “advance care planning” (cluster #1), and “inflammation” (cluster #2). Based on the distribution of keyword clusters, our findings suggest that advanced lung cancer, treatment modalities, and inflammation are prominent research areas within the domain of depression in lung cancer. Furthermore, fluoxetine administration and aerobic exercise have emerged as popular therapeutic interventions for managing depression comorbid with lung cancer. This indicates an increasing concern among researchers regarding depression in lung cancer patients and a search for treatments to alleviate their symptoms.

According to **Figure 9B**, “advanced care planning” (cluster #1), “advanced cancer” (cluster #3), and “breathlessness” (cluster #5) almost cover the entire timeline, indicating that they have consistently been key topics in the field. The emergence of “fluoxetine” and “aerobic exercise” after 2015 indicates a growing interest among researchers in addressing depression related to lung cancer treatment. However, the available treatment options remain limited.

## 4 Discussion

As the precise etiology of lung cancer-related depression remains elusive and treatment options are limited, a comprehensive scientific analysis of literature pertaining to this field may offer novel insights for both research and treatment of depression in lung cancer patients. Therefore, this study employed a bibliometric approach suited for evaluating extensive scientific literature within a specific research domain. By utilizing the WoSCC database to comprehensively review relevant literature from 2014 to 2024 pertaining to depression in lung cancer, a bibliometric analysis was conducted to assess research hotspots, and potential trends, and provide reference guidelines for future researchers. The ensuing subsections will delve into a comprehensive discussion of the primary findings derived from this analysis.

### 4.1 Key findings

#### 4.1.1 General information

In the previous decade, the investigation of depression in the context of lung cancer has witnessed a phase of accelerated progress, as evidenced by the consistent almost annual growth in the quantity of relevant scholarly publications from 2014 to 2024. This analysis implies a gradual increase in researchers’ attention toward lung cancer-related depression.

China, the United States, England, Canada, and Germany prominently emerge as the primary regions for publishing articles on lung cancer-related depression. Notably, the United States not only demonstrates a substantial volume of publications but also maintains robust international collaborations, thereby solidifying its prominent position as an academic leader in this field. China leads in terms of publication volume, however, it lacks robust international collaborations and its scholarly impact remains comparatively lower than that of the United States, England, and Canada. This situation may serve as a catalyst for Chinese researchers to produce literature of higher quality, and foster enhanced international cooperation.

Harvard University has consistently demonstrated its commitment to lung cancer-related depression research through the highest number of publications and robust collaborations with other institutions, establishing significant influence in the field of research. It is noteworthy that while there exists a certain degree of collaboration between universities and healthcare organizations, the collaborations between institutions remain limited. Additionally, the top 10 institutions in terms of publication volume predominantly originate from the United States, with Harvard University being among them. Consequently, we advocate for researchers to enhance inter-institutional collaboration, particularly with American institutions.

Publications serve as conduits for disseminating research achievements, and effective scientific communication necessitates the dissemination of research results in internationally recognized peer-reviewed journals. Therefore, through an analysis of journal sources, researchers can quickly identify the journals most suitable for their papers. Among them, Psycho-oncology and Supportive care in cancer are the two leading journals in terms of publication volume, both surpassing 40 publications, which is significantly higher compared to other journals. This indicates that these journals prioritize research in this field, and scholars in the field can give priority to publishing their findings in these journals.

Journal of Clinical Oncology was the most cited. The majority of the journals, whether they are productive or co-cited, pertain to the field of oncology, while a minority of them are related to psychology. Furthermore, it is noteworthy that Psycho-Oncology stands as the sole journal exclusively dedicated to exploring the psychological well-being of individuals affected by cancer. This observation implies that current concerns regarding depression in lung cancer patients primarily stem from oncology researchers; however, an increasing number of scholars, including those specializing in psychology, are now directing their attention toward understanding the psychological state of individuals with cancer. Interestingly, the USA and England journals had the largest share in terms of both the top 10 productive journals and the top 10 co-cited journals, indicating that USA and England journals exert significant influence in this field.

#### 4.1.2 Evolution of research hotspots and frontiers

The selection of keywords in a paper is a meticulous process aimed at accurately representing the topic and core content. The co-occurrence relationship among keywords, clusters formed by their co-occurrence, and bursts all play crucial roles as indicators reflecting current hot topics and development trends within a specific research field. Additionally, an analysis of co-cited literature can effectively reveal the intellectual structure and thematic clusters (21). Based on the analysis of keywords and co-cited references, the research on depression related to lung cancer can be broadly categorized into the following aspects, highlighting current hotspots and frontiers.

The co-occurrence of keywords and the co-cited references analysis indicate that the field of research on the epidemiology of lung cancer-related depression is a hotspot. Furthermore, it is worth noting that comorbid mental disorders, especially anxiety, are commonly observed among patients with lung cancer.

The prevalence of lung cancer-related depression ranges from 11% to 71.7%. Variations in geographic regions, study populations, and sample sizes have contributed to the diverse findings regarding morbidity (5, 7, 29). Given the high occurrence of depression in lung cancer patients and its detrimental effects on their well-being, considerable attention has been directed towards identifying risk factors contributing to the development of depression in this population. Several studies have investigated the risk factors associated with depression in lung cancer patients, including gender, hypertension, diabetes mellitus, postoperative complications, and TNM staging (2, 30, 31). In addition, the individual factors of patients play a crucial role in determining the mental health status of lung cancer patients. A study utilizing a standardized questionnaire revealed that lung cancer patients who required assistance with hospital procedures, daily living activities, and self-care exhibited significantly higher levels of anxiety and depression compared to those without such needs. This finding suggests that the assistance requirements encountered by lung cancer patients in their daily lives may be an important contributing factor to their depressive symptoms (32). Furthermore, another study employing a questionnaire-based approach demonstrated that the influence of social support on depression varied significantly across different stages of lung cancer. Specifically, early-stage lung cancer patients were more inclined to depend on direct social support for alleviating their depressive symptoms, whereas advanced-stage lung cancer patients experienced relatively weaker effects from their own social support on depression (33). However, the findings from these studies currently remain contentious; for instance, the association between gender and lung cancer-related depression as a risk factor remains inconclusive (30, 31). Furthermore, research has indicated that lung cancer patients exhibit a higher prevalence of depression compared to individuals with other types of cancer (7), potentially attributed to the elevated mortality rate and diminished quality of life associated with lung cancer. These findings highlight the imperative for healthcare professionals to prioritize the identification and examination of risk factors that contribute to depression in lung cancer patients. Individual variables, such as the stage of disease and specific patient complaints, should also be considered to provide robust and comprehensive evidence for effective prevention and treatment strategies within clinical practice.

The prevalence of depression among lung cancer patients should not be overlooked, as its detrimental effects, particularly the significant impairment of patients’ quality of life, warrant considerable attention. The keyword “quality of life” exhibits the highest frequency, while three out of the top 10 co-cited count references are specifically related to quality of life. This indicates that the impact of depression on the quality of life among lung cancer patients represents a hot.

The study entitled “Emotional Problems, Quality of Life, and Symptom Burden in Lung Cancer Patients”, authored by Eleshia J. Morrison et, al, has been identified as the fourth most frequently cited publication. It posits that a diminished quality of life significantly contributes to the onset of depression in lung cancer patients (34). Relevant research conducted by other scholars has corroborated the finding (35). Patients’ depressive symptoms, pain, and fatigue can significantly diminish their quality of life (36). Moreover, depressive symptoms can directly impact patients’ emotional states and cognitive functions, increasing the likelihood of feelings such as helplessness, anxiety, and hopelessness—psychological burdens that further compromise the quality of life (37). Importantly, these depressive symptoms may lead patients to withdraw from social activities, adversely affecting their relationships with family and friends. This reduction in social engagement diminishes the level of social support available to them and exacerbates the decline in quality of life (38). Additionally, a study indicated that lung cancer patients experiencing major depression often perceive their condition as uncontrollable by treatment and report feelings of hopelessness. Such perceptions may contribute to lower survival rates among lung cancer patients (10). Therefore, while depression is merely one symptom experienced by lung cancer patients, it warrants serious attention from healthcare providers.

According to **Figures 9A and B**, “inflammation” (cluster #2) covers almost the entire timeline, indicating that the association between inflammation and depression in lung cancer patients is another significant area of interest.

Inflammation is associated with the development of lung cancer (39) and may contribute to the development of depression in lung cancer patients through various mechanisms. One study discovered that a high level of tumor mutational load represents a somatic biological parameter related to cancer, which could potentially influence depressive symptoms by inducing inflammation or other pathways (40). Patients with depression often exhibit dysregulated levels of inflammatory markers; for instance, those with metastatic lung cancer combined with depression may have elevated C-reactive protein (CRP) levels and an increased CRP/albumin ratio (41, 42). Furthermore, chemotherapy can trigger the release of pro-inflammatory cytokines, leading to alterations in neurotransmitters and behavioral manifestations. Consequently, anxiety and depression frequently manifest in lung cancer patients undergoing chemotherapy (43). Another study found that inflammation combined with depression was a risk factor for poor survival in lung cancer (44). It is evident that there exists a reciprocal relationship between inflammation and depression in lung cancer patients, both of which exert detrimental effects on the overall well-being of individuals with lung cancer. Although the precise mechanism underlying the involvement of inflammation in depression associated with lung cancer remains elusive, these current findings introduce novel perspectives to investigating depressive symptoms in lung cancer patients.

The treatment of individuals with comorbid lung cancer and depression has been the primary focus of researchers. Based on the reference cited and keyword analysis conducted in this study, early palliative care, exercise therapy, psychological support, and fluoxetine are widely recognized as effective treatment modalities for those with concurrent lung cancer and depression.

Fluoxetine hydrochloride is a widely utilized antidepressant in clinical practice and has been demonstrated to reduce overall mortality among cancer patients (45). Research studies have indicated that chronic stress promotes the growth of cancer cells, while fluoxetine may exert its inhibitory effects on tumor growth by suppressing the kynurenine pathway and enhancing cellular immunity (14). Furthermore, an additional study revealed that fluoxetine hinders lung cancer progression through activation of the ATF4-AKT-mTOR signaling pathway, inducing cell cycle arrest and autophagy to impede cancer cell proliferation without affecting normal lung epithelial cell proliferation (46). However, further evidence-based research is necessary to establish the safety and efficacy of fluoxetine as a viable clinical treatment strategy for patients with lung cancer-related depression.

The integration of palliative care into oncology practice has garnered substantial evidence in support, particularly for advanced cancer patients (47, 48). A total of three articles in the References with the Strongest Citation Bursts literature emphasizing the benefits of early palliative care for patients with lung cancer-related depression were identified. Two of these articles appeared in both the highly cited literature and References with the Strongest Citation Bursts literature (26, 27, 49). Considering the heterogeneity in demographic characteristics and cultural practices across diverse regions, it is crucial to investigate region-specific palliative care approaches tailored specifically for lung cancer populations. For instance, the E-warm model, characterized by distinct Chinese attributes, represents a unique and comprehensive palliative care approach. Recent studies have provided evidence that this model leads to improved quality of life, prolonged survival, enhanced psychological well-being, reduced pain levels, and increased nutritional satisfaction among patients with non-small cell lung cancer (50). However, a significant disparity exists between the development of cancer-related palliative care in China and international standards, while limited research investigates diverse palliative care models across different regions. This emerging area may become a focal point for early palliative care targeting lung cancer-related depression in the future.

Exercise therapy, as a non-pharmacological intervention, demonstrates considerable potential in the treatment of lung cancer patients experiencing depression. A study indicates that an eight-week regimen of Baduanjin exercise can significantly alleviate anxiety and depression while enhancing the quality of life for lung cancer patients (51). A twelve-week program of partner-supported yoga not only improves the psychological well-being of these patients but also enhances their physical functioning (52). Additionally, Taijiquan, another gentle form of exercise, has demonstrated positive outcomes in several studies. A randomized controlled trial revealed that tai chi effectively reduces anxiety and depression among lung cancer patients while improving exercise endurance and cardiopulmonary function (53). Moreover, a meta-analysis highlighted that preoperative exercise training significantly mitigates the risk of postoperative complications in lung cancer surgery—a finding crucial for facilitating postoperative recovery (16). Several studies have also underscored the advantages of regular and periodic exercise for lung cancer patients, extending beyond merely psychological benefits (54, 55). Furthermore, exercise therapy exhibits a minimal incidence of adverse events (56), thus warranting further comprehensive investigation as an economical treatment modality with limited side effects.

Psychotherapy plays a crucial role in the clinical management of lung cancer patients experiencing depression. Cognitive Behavioral Therapy (CBT) is among the most extensively researched psychological interventions, demonstrating significant improvements in symptoms of depression and anxiety by assisting patients in identifying and modifying negative thought patterns (57). Research indicates that lung cancer patients who undergo CBT not only experience alleviation of depressive symptoms but also achieve enhancements in their overall quality of life (58). Furthermore, Mindfulness-Based Stress Reduction (MBSR) effectively mitigates excessive concerns regarding illness and future uncertainties by guiding patients to concentrate on the present moment. A meta-analysis has suggested that MBSR may be beneficial in reducing anxiety, depression, and fatigue among lung cancer patients. Nevertheless, it is important to note that the overall quality of evidence remains low; thus, more rigorous studies are warranted for further validation (59). It is worth noting that psychotherapy extends beyond individual interventions for lung cancer patients; it also encompasses multidimensional support strategies, such as family support (60) and group interventions (61). These approaches can significantly enhance the psychological resilience of patients, thereby improving their overall health. Furthermore, the application strategies of psychotherapy differ according to the various stages of cancer progression. For instance, during the early diagnostic phase, psychological interventions may serve to prevent the emergence of depressive symptoms. In contrast, in the advanced stages of the disease, greater emphasis is placed on alleviating existing depressive symptoms to enhance patients’ quality of life (62).

### 4.2 Research gap and future directions

In the field of lung cancer-related depression research, an analysis from multiple perspectives—including the number of national publications, authors, cited authors, journals, and cited journals—reveals that the scientific research influence of certain non-Western countries is significantly inferior to that of developed nations such as the United States and the United Kingdom. Furthermore, there exists a notable lack of international collaboration. Several factors probably contribute to this disparity: insufficient investment in scientific research funding limits both the depth and breadth of studies; a weak cooperation network hinders integration into the global academic community; a shortage of skilled researchers restricts advancements in scientific inquiry; and language barriers impede effective dissemination of findings as well as collaborative efforts. To address these challenges, it is recommended that non-Western countries increase their investment in research funding and enhance training programs for researchers aimed at improving English language proficiency when economically feasible. Additionally, researchers should actively engage in international academic conferences and proactively present their work to foster international collaboration and elevate the impact of their scientific contributions.

In addition, the integration of the hotspot analysis from this study with relevant literature reveals that several issues remain to be addressed in the realm of lung cancer-related depression.

Firstly, there exists considerable variability in the reported incidence of depression among lung cancer patients. The findings of various studies may be influenced by factors such as geographic location, study populations, and sample sizes, resulting in inconsistent conclusions. Furthermore, while numerous studies have investigated a range of risk factors, the relationships among some of these factors remain ambiguous; for instance, consensus has yet to be reached regarding the association between gender and depression. To tackle these challenges effectively, it is imperative that research standards are standardized moving forward. Additionally, multicenter studies with large sample sizes and cross-cultural perspectives should be conducted to minimize bias and enhance the generalizability of results.

Secondly, although studies have been conducted to elucidate the role of inflammation in depression among lung cancer patients, several issues remain unresolved. The molecular mechanisms through which inflammation mediates depression in this population are not yet understood. Furthermore, most clinical studies are cross-sectional in design and lack longitudinal cohort studies necessary to establish causality. Additionally, there is a deficiency of standardized methods for detecting inflammation markers. In the future, it will be essential to establish *in situ* animal models of lung cancer and conduct multicenter prospective cohort studies. Standardization of testing protocols should also be prioritized. Moreover, integrating oncology, psychology, and immunology will be crucial for advancing research progress in this field.

Thirdly, although exercise therapy and psychotherapy demonstrate therapeutic potential for patients with depression associated with lung cancer, the majority of studies are characterized by small sample sizes, predominantly short-term observations, and a limited number of related meta-analyses. Therefore, there is a pressing need for more large-scale, multicenter randomized controlled trials (RCTs) in the future to validate the long-term effects of these non-pharmacological interventions. Furthermore, it is advisable to investigate the standardized implementation and individualized adaptation of psychotherapies further, particularly their applicability among patients from diverse cultural backgrounds and at various stages of disease progression.

Finally, the treatment options for depression associated with lung cancer remain limited. This limitation may stem from an insufficient understanding of the co-morbid mechanisms of the disease, a lack of interdisciplinary collaboration, and the absence of a standardized assessment system. From the patient’s perspective, the complexity of the treatment process and the significant psychological burden further impede the effective management of depression. To address these research gaps in the future, it is essential to strengthen multidisciplinary collaboration, establish standardized assessment protocols, and develop novel therapeutic approaches. In clinical practice, optimizing patient-centered treatment pathways will be crucial to providing more comprehensive and effective care for patients suffering from both lung cancer and depression.

### 4.3 Strengths and weaknesses

Our study possesses several notable strengths. Firstly, it presents a comprehensive analysis of the research focal points, emerging trends, and seminal publications related to depression in lung cancer over the past decade for the first time. Secondly, bibliometric analysis offers a more comprehensive and visually accessible approach compared to traditional literature reviews.

Furthermore, bibliometric studies inherently possess certain limitations. Firstly, the study exclusively utilized WoSCC, which is widely recognized as the most authoritative and comprehensive database in bibliometric research. This deliberate selection may inadvertently exclude literature that is not encompassed by this particular database. To address this gap, our future research may explore the integration of additional databases, such as PubMed and Scopus, in order to offer a more comprehensive perspective on the research. Additionally, to capture prevailing research trends and focus on cutting-edge topics, this study solely concentrated on literature published within the last decade to ensure currency.

## 5 Conclusion

Our study presents a comprehensive bibliometric analysis of the knowledge structure and emerging frontiers in lung cancer-related depression research from 2014 to 2024, marking the first endeavor of its kind in this domain. Lung cancer-related depression has garnered increasing attention from researchers, as evidenced by the growing number of publications each year.

In summary, China exhibits a significant volume of publications; however, the United States and England demonstrate a greater impact on research. Fostering international collaboration among research institutions and scholars from diverse countries is imperative, particularly in the case of China. Jennifer S. Temel emerges as a highly influential author. The journal with the highest number of publications is Supportive Care in Cancer, while the Journal of Clinical Oncology has garnered the most citations.

Our study identifies the epidemiology of lung cancer-associated depression, the deleterious effects of depression on lung cancer patients, the complex relationship between inflammation and depression in lung cancer patients, and the exploration of treatments for lung cancer-associated depression as hot topics in the field. Based on these findings, it is recommended that researchers delve deeper into the underlying pathological mechanisms of lung cancer-related depression to establish a theoretical foundation for developing novel therapies. Policymakers should prioritize mental health considerations for lung cancer patients by formulating and implementing relevant policies aimed at enhancing the availability and accessibility of psychological interventions. Furthermore, clinical practitioners ought to enhance early screening processes and provide individualized interventions by integrating multidisciplinary knowledge from oncology, psychology, and immunology to deliver comprehensive treatment plans tailored to each patient.

## Data Availability

Publicly available datasets were analyzed in this study. This data can be found here: http://webofscience.com.
