# Supplementary material for: Global research and emerging trends in depression in lung cancer: a bibliometric and visualized study from 2014 to 2024
Source: Front Oncol. 2025 Mar 5;15:1490108. doi: 10.3389/fonc.2025.1490108 (PMC11919658; doi:10.3389/fonc.2025.1490108)
Supplement: Supplementary file 1 [file DataSheet1.docx]

Supplementary Material

# Supplementary Figures and Tables

## Supplementary Figures

**
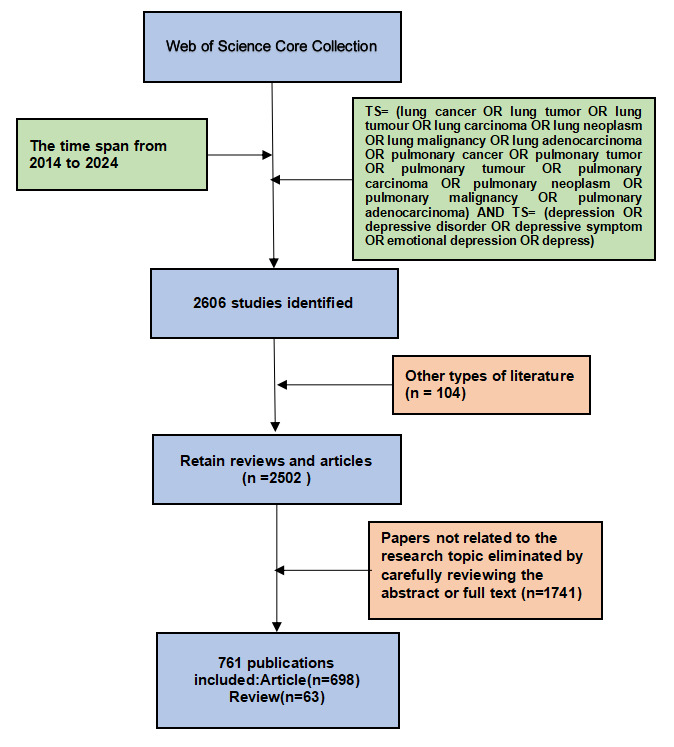
**

**Figure 1.** The implementation process of the study. (Supplementary_Material)

**
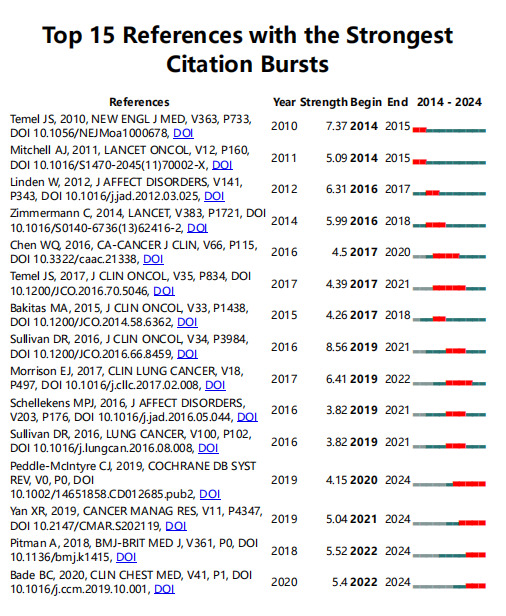
**

**Figure 7.** Top 15 references with the strongest citation bursts.(Supplementary_Material)

## Supplementary Tables

| Terms | Terminology Explanation |
| --- | --- |
| Node Size | The size of a node typically reflects its influence or significance within a given context (e.g., literature, author, institution, etc.), which is often correlated with the number of citations or the frequency of occurrence in the literature. A larger node indicates a higher frequency of occurrence and greater impact within the research domain. |
| Node Color | The color of nodes generally signifies different time periods or clusters, among other information. Variations in color can assist in distinguishing the publication year of the literature or identifying specific research directions. |
| The thickness of Lines Between Nodes | The thickness of lines connecting nodes represents the strength of their relationships; thicker lines indicate stronger connections between two nodes. |
| Red Line Segments in Burst Analysis | In the context of emergence analysis, red line segments signify a rapid growth or sudden increase in a research topic or keyword over a defined time period. This typically indicates an emerging trend within the field. |
| Blue Line Segments in Burst Analysis | Blue line segments represent the stability or persistence of a research topic or keyword, reflecting a long-term trend of sustained interest within the discipline. |
| Intensity of Burst Analysis | The intensity metric denotes the degree of emergence associated with a research topic or keyword during a specific timeframe; higher intensity levels correspond to greater research fervor and interest in the subject matter over that brief period. |
| Centrality | Centrality measures the significance of a node within a network. A high centrality score indicates that the node occupies a pivotal position in the research network, typically corresponding to a literature or research area with substantial influence. |
| Modularity Value (Q Value) | The modularity value reflects the extent of connectivity among different subgroups (modules) within the network. A higher Q value signifies clearer delineation between subgroups and suggests a more rational partitioning of modules in the network. |
| Average Silhouette Value (S Value): | The average silhouette coefficient is employed to assess clustering quality. The closer the S value approaches 1, the more effective the clustering outcome becomes, indicating greater cohesion within clusters and enhanced differentiation between them. |
